# Case Report: a case of prolonged post-traumatic duodenal obstruction complicated by traumatic pancreatitis and Wernicke's encephalopathy: a multidisciplinary challenge

**DOI:** 10.3389/fmed.2026.1849748

**Published:** 2026-06-12

**Authors:** Li-Bin Deng, Wen-Na Liu, Zheng-Hong Jiang, Li-Hao Deng

**Affiliations:** 1 Department of Gastrointestinal Hernia and Abdominal Wall Surgery, Affiliated Hospital of Chengdu University, Chengdu, Sichuan, China; 2 Department of Laboratory Medicine and Sichuan Provincial Key Laboratory for Human Disease Gene Study, Sichuan Provincial People's Hospital, School of Medicine, University of Electronic Science and Technology of China, Chengdu, China

**Keywords:** combined abscess (retroperitoneal abscess and intra-abdominal abscess), duodenal obstruction, multidisciplinary care, post-traumatic, thiamine deficiency, traumatic pancreatitis, Wernicke's encephalopathy

## Abstract

**Background:**

Post-traumatic gastrointestinal injuries, especially those involving the duodenum and proximal jejunum, present significant management challenges. Complications such as retroperitoneal abscess, high intestinal obstruction, and secondary pancreatitis can lead to prolonged fasting and severe nutritional deficiencies. We report a complex case where a patient developed a rare triad of post-operative combined abscess (retroperitoneal abscess and intra-abdominal abscess)-induced high intestinal obstruction, traumatic pancreatitis, and subsequent Wernicke's encephalopathy (WE), highlighting the diagnostic and therapeutic complexities.

**Case presentation:**

A 52-year-old woman was admitted 10 days after undergoing a partial jejunectomy for a traumatic jejunal perforation. Post-admission, she developed a combined abscess (retroperitoneal abscess and intra-abdominal abscess) adjacent to the duodenum, causing high intestinal obstruction manifested by intractable nausea and vomiting. Despite conservative management and placement of a nasointestinal decompression tube, her symptoms persisted. The clinical course was further complicated by a significant elevation in serum lipase, indicating traumatic pancreatitis. Following prolonged fasting and vomiting, the patient received total parenteral nutrition (TPN) during the prolonged period of duodenal obstruction; however, the thiamine content in the TPN formulation was inadequate, contributing to the development of thiamine deficiency. The patient presented with atypical neurological symptoms, including severe dizziness, orthostatic instability, and nystagmus (horizontal and vertical). Brain MRI revealed characteristic hyperintensities in the periaqueductal gray matter, mammillary bodies, and bilateral medial thalami, confirming a diagnosis of Wernicke's encephalopathy. High-dose parenteral thiamine (vitamin B1) was promptly initiated, resulting in rapid and significant improvement in both neurological and gastrointestinal symptoms. Subsequently, the combined abscess (retroperitoneal abscess and intra-abdominal abscess) resolved, duodenal patency was confirmed by imaging, and the patient successfully transitioned to an oral diet.

**Conclusion:**

This case underscores the critical importance of a multidisciplinary approach in managing complex post-traumatic gastrointestinal sequelae. It highlights the need for a high index of suspicion for Wernicke's encephalopathy in patients with prolonged fasting, especially when neurological symptoms are atypical. Early recognition and treatment with thiamine are paramount to prevent irreversible neurological damage. Furthermore, the interplay between mechanical obstruction, inflammation (pancreatitis and abscess formation), and metabolic derangements (thiamine deficiency) created a self-perpetuating cycle of morbidity that required sequential, targeted interventions.

## Introduction

Traumatic injuries to the small bowel and mesentery, though less common than solid organ injuries, carry significant morbidity and mortality. The management of injuries to the proximal jejunum is further complicated by its anatomical proximity to the duodenum and pancreas. Post-operative complications such as intra-abdominal abscesses, fistulas, and prolonged ileus are well-documented. High intestinal obstruction, particularly in the region of the duodenum and proximal jejunum, can lead to a cascade of metabolic and neurological sequelae.

Wernicke's encephalopathy (WE), an acute neuropsychiatric condition resulting from thiamine (vitamin B1) deficiency, is classically associated with chronic alcoholism. However, it is an under-recognized and critical complication in non-alcoholic patients with prolonged fasting, hyperemesis, or malabsorptive states, such as those following major gastrointestinal surgery. The classic triad of encephalopathy, oculomotor dysfunction, and gait ataxia is present in only a minority of cases, often leading to delayed diagnosis and potentially irreversible Korsakoff syndrome. This case report presents a patient who, following a traumatic jejunal injury, developed a complex interplay of combined abscess (retroperitoneal abscess and intra-abdominal abscess), high intestinal obstruction, traumatic pancreatitis, and ultimately, an atypical presentation of WE. We discuss the diagnostic challenges and the successful multidisciplinary management that led to a favorable outcome.

## Case presentation

A 52-year-old woman was transferred to our institution on 9 October 2025 with a chief complaint of persistent symptoms 10 days following an external surgical intervention. Her initial surgery, performed at an outside hospital, was a partial jejunectomy for a traumatic jejunal perforation located approximately 10 cm distal to the ligament of Treitz. Vital signs on initial presentation to our hospital: blood pressure: 128/76 mmHg, heart rate: 92 beats per minute, respiratory rate: 18 breaths per minute, body temperature: 37.8 °C, and oxygen saturation: 98% on room air. Laboratory findings: white blood cell count: 8.4 × 10^9^/L, C-reactive protein: 86 mg/L, serum lipase: 42 U/L (reference range < 60 U/L), and serum amylase: 89 U/L (reference range < 100 U/L). Past medical history: The patient had no significant past medical history, including no history of chronic alcoholism, malnutrition, or prior gastrointestinal disorders.

Upon admission, an abdominal computed tomography (CT) scan revealed a combined abscess in close proximity to the duodenum (Figure 1). The patient was experiencing recurrent nausea and vomiting, suggestive of a high intestinal obstruction secondary to the abscess. Initial management consisted of nasogastric tube decompression, bowel rest, fluid resuscitation, broad-spectrum antibiotics, acid suppression, and parenteral nutrition. Upon diagnosis of duodenal obstruction, the patient was placed on bowel rest and started on TPN. Notably, the TPN formulation contained suboptimal thiamine supplementation, which, combined with prolonged fasting, placed the patient at increased risk of thiamine deficiency. Despite these measures, her symptoms persisted.

**Figure 1:**
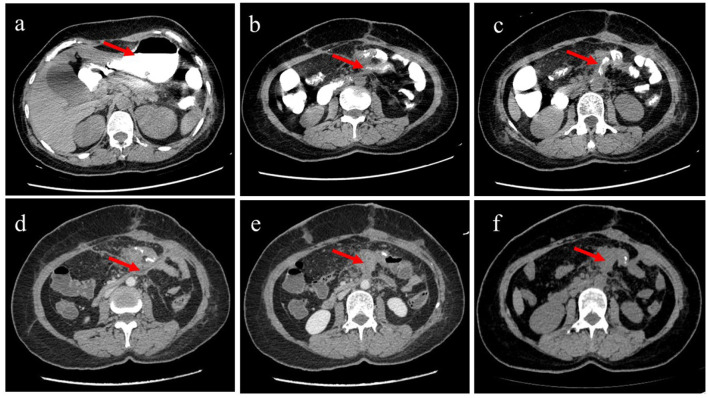
CT findings of delayed gastric emptying and mixed abscess formation. **(a–c)** Axial contrast-enhanced CT images obtained 6 h after the oral administration of iohexol show marked retention of the contrast agent within the gastric lumen and slow, delayed passage through the duodenum, indicating partial duodenal obstruction. **(d–f)** Axial CT images demonstrate a mixed retroperitoneal and intra-abdominal abscess located around the horizontal portion of the duodenum (retroperitoneal component) and adjacent to the ligament of Treitz and the proximal jejunum (intraperitoneal component).

On 5 November 2025, a nasointestinal obstruction tube was placed endoscopically to facilitate decompression beyond the ligament of Treitz (Figure 2). However, she continued to experience nausea and vomiting, even with tube irrigation. On 23 November 2025, serum lipase was found to be significantly elevated at 768 U/L (reference range < 60 U/L), leading to a diagnosis of traumatic pancreatitis, a likely consequence of the initial trauma or the adjacent inflammatory process.

**Figure 2:**
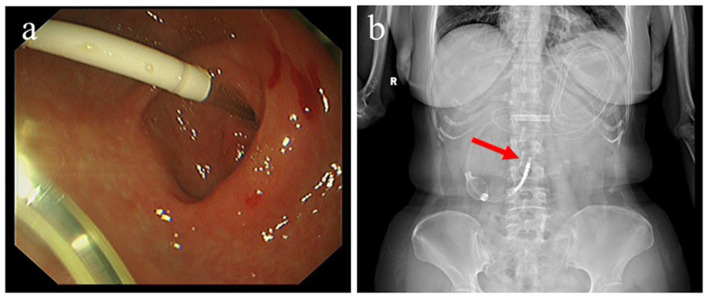
Placement and positional confirmation of the nasointestinal obstruction tube. **(a)** Endoscopic image shows the intraoperative placement of a nasointestinal obstruction tube. **(b)** Follow-up abdominal plain radiograph demonstrates the tube positioned at the horizontal portion of the duodenum (arrows).

The clinical course was further complicated by the onset of neurological symptoms on 25 November 2025. The patient reported severe generalized weakness, palpitations, and dizziness. Concurrently, she remained hypotensive (87/56 mmHg) and tachycardic (139 bpm), with no response to metoprolol. Two days later, she developed acute vertigo and gait instability. A neurological examination revealed horizontal and vertical nystagmus and a positive Romberg sign. There were no focal motor or sensory deficits.

A brain MRI with DWI performed on 27 November 2025 showed classic findings of WE: hyperintense signal changes in T2-weighted and FLAIR sequences in the periaqueductal gray matter, the mammillary bodies, and the medial thalami bilaterally (Figure 3). No acute infarction was noted.

**Figure 3:**
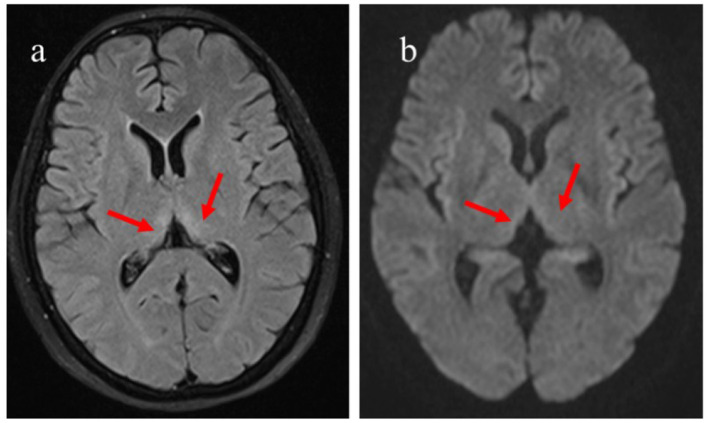
Comparison of brain MRI before and after treatment. **(a)** Pre-treatment axial T1-weighted and T2-weighted images demonstrate symmetrically distributed nodular lesions with iso-to-long T1 and long T2 signal intensity involving the periaqueductal region, mammillary bodies, and bilateral medial thalami (arrows). **(b)** Follow-up MRI after treatment shows complete resolution of these symmetrical nodular lesions in the abovementioned regions (arrows).

The patient was immediately started on high-dose parenteral thiamine [200 mg three times daily (TID) intramuscularly] and other water-soluble vitamins. The response was dramatic. Her nausea and vomiting improved considerably, and she was able to tolerate small amounts of oral intake without symptoms.

A follow-up gastrointestinal series on 19 December 2025 demonstrated delayed gastric emptying but confirmed patency of the duodenum and proximal jejunum, with no evidence of obstruction. A subsequent abdominal CT with oral contrast showed near-complete resolution of the combined abscess and a marked reduction in surgical site edema (Figure 4). The patient's clinical condition stabilized, and she was successfully transitioned to a semi-solid diet without nausea, vomiting, or abdominal pain. She was discharged on a regimen of oral thiamine and close follow-up with gastroenterology and neurology.

**Figure 4:**
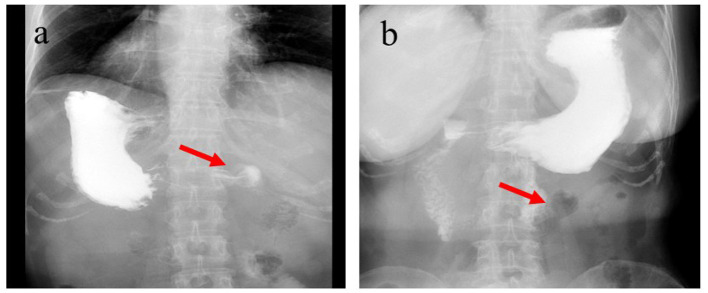
Dynamic radiographic imaging after oral contrast administration. **(a, b)** Sequential dynamic radiographs demonstrate smooth passage of the contrast medium through the duodenum and into the proximal jejunum, without evidence of obstruction or delay (arrows indicate smooth passage of contrast medium through the duodenum and into the jejunum).

Clinical timeline of the patient's disease course, including traumatic injury, surgical intervention, diagnosis of complications, medical treatment, and 6-month follow-up outcomes.

## Follow-up and outcomes

At the 6-month follow-up, the patient remained completely asymptomatic with no neurological sequelae. She had fully resumed normal daily activities and maintained an unrestricted oral diet. No neurological or gastrointestinal recurrence was observed during follow-up, and the patient's quality of life had returned to baseline. She will continue regular neurological follow-up to monitor for potential late-onset sequelae.

## Discussion

This case illustrates a cascade of post-traumatic complications (Table 1)—combined retroperitoneal and intra-abdominal abscess, high intestinal obstruction, traumatic pancreatitis, and Wernicke's encephalopathy (WE)—and demonstrates the effectiveness of sequential, multidisciplinary management (1).

**Table 1:** Clinical timeline of the patient's disease course.

Time point	Key events
30 September 2025	Abdominal trauma; traumatic jejunal perforation
30 September 2025	Partial jejunectomy performed at an outside hospital
9 October 2025 Post-operative day 10	Transferred to our hospital, diagnosed with combined abscess and partial duodenal obstruction; antibiotics and TPN initiated
5 November 2025 Post-operative day 37	Endoscopic nasointestinal decompression tube placement
23 November 2025 Post-operative day 55	Traumatic pancreatitis diagnosed
25 November 2025 Post-operative day 57	Atypical neurological symptoms occurred
27 November 2025 Post-operative day 59	Wernicke's encephalopathy diagnosed; high-dose thiamine started
19 December 2025 Post-operative day 71	Abscess resolved; duodenal patency confirmed; oral diet resumed
6-month follow-up	Asymptomatic; complete recovery without sequelae

Combined abscess as a cause of high jejunal obstruction and pancreatic injury from blunt abdominal trauma is notoriously difficult to diagnose (2–5). The pancreas is retroperitoneal, and injuries are often subtle and easily missed, especially in polytrauma (6, 7). Conventional imaging frequently fails to detect subtle pancreatic injuries, and post-traumatic pancreatitis may not show radiographic changes for hours (8). Isolated pancreatic injuries from blunt trauma present with delayed symptoms for at least 24–48 h, with a mean diagnostic delay of 28 days (range 4–60 days) (9). In our patient, the initial surgery was performed for traumatic jejunal perforation. However, the retroperitoneal findings (pancreatitis and retroperitoneal abscess) were most likely attributable to a missed small pancreatic laceration or contusion sustained at the time of the original trauma, rather than a complication of the jejunal repair. The delayed symptom onset is consistent with the natural history of traumatic pancreatitis. Six hours after oral iohexol, abdominal CT showed gastric contrast retention. The patient had recurrent nausea and vomiting with delayed duodenal contrast passage, indicating partial duodenal obstruction. The combined retroperitoneal and intra-abdominal abscess was managed conservatively with intravenous antibiotics and supportive care. The decision to adopt conservative management rather than percutaneous or surgical drainage was based on multidisciplinary discussion involving gastrointestinal surgery, gastroenterology, interventional radiology, and infectious disease specialists. The abscess was small (approximately 3 cm), and the patient remained hemodynamically stable without persistent sepsis. Serial imaging demonstrated gradual resolution under antibiotic therapy alone. Although percutaneous drainage is the first-line intervention for most retroperitoneal abscesses, it carries risks, including bleeding, infection, and catheter-related complications. In this clinically stable patient with a small, treatment-responsive lesion, the risks of invasive procedures were considered to outweigh the benefits. Moreover, the duodenal obstruction was related not only to mechanical compression but also to surrounding inflammation and edema, which improved gradually with time and conservative treatment. Upper endoscopy was not performed because the patient remained hemodynamically stable with no hematemesis or melena. Duodenal obstruction was diagnosed based on clinical manifestations (persistent nausea, vomiting, and intolerance to oral intake) and radiologic findings (delayed contrast passage through the duodenum). Although endoscopy could provide direct visualization, it was not considered necessary given the satisfactory response to conservative management. The combined abscess is a known but challenging complication after surgery for penetrating or blunt trauma to the upper gastrointestinal tract (10, 11). In the present patient, the abscess was located adjacent to the duodenum and proximal jejunum, causing extrinsic compression and functional obstruction. Despite adequate drainage (via a nasointestinal tube) and antibiotic therapy (12–15), the obstruction persisted for approximately 2 months because of the anatomical location and associated inflammation. Resolution of the abscess was confirmed only after thiamine treatment and time-dependent healing, highlighting that such obstruction may require patience and supportive care rather than early surgical re-intervention (16). Traumatic pancreatitis was an important comorbidity in this case; its development (lipase 768 U/L) added another layer of complexity. It likely resulted from direct contusion during the initial injury or from the adjacent inflammatory process (17). The pancreatitis further delayed the return of bowel function, exacerbated nutritional deprivation, and increased the metabolic demand for thiamine (18, 19).

Typical presentations and early MRI play a very critical role in identifying Wernicke's encephalopathy. The most important lesson is the recognition of WE in a non-alcoholic patient with prolonged fasting. The patient had been on TPN for >6 weeks (20–22), but standard TPN contains only a minimal amount of thiamine (≈3–5 mg/day), which is insufficient for patients with hyperemesis, infection, and increased metabolic demands. The classic triad (consciousness disturbance, oculomotor dysfunction, ataxia) was incomplete: The patient was alert but had nystagmus, ataxia (positive Romberg sign), and autonomic dysfunction (hypotension, tachycardia) (23, 24). Such atypical presentations are common in non-alcoholic WE and frequently lead to missed or delayed diagnosis. The initial symptoms of severe weakness, dizziness, and refractory tachycardia were mistaken for a cardiac or autonomic issue (25); the lack of response to metoprolol was a clue (24, 26). Subsequent vertigo and nystagmus prompted brain MRI, which revealed pathognomonic symmetrical hyperintensities in the periaqueductal area, mammillary bodies, and medial thalami—findings with high sensitivity and specificity for WE (16, 26–28). Thiamine levels (TPP/TDP) were not measured because this assay was not available at our institution. However, the diagnosis of WE was firmly established based on clinical presentation and characteristic MRI findings. Prompt high-dose parenteral thiamine [200 mg three times daily (TID) intramuscularly, plus other water-soluble vitamins] led to rapid improvement in both gastrointestinal and neurological symptoms (23, 29). Notably, nausea and vomiting—initially attributed solely to mechanical obstruction—improved significantly after thiamine administration, suggesting that autonomic dysfunction and thiamine deficiency themselves can contribute to gastroparesis. The patient's eventual full neurological recovery underscores the importance of early treatment (30).

WE following gastrointestinal surgery or prolonged fasting remains underdiagnosed. Cases have been reported after bariatric surgery, gastric cancer surgery, and intestinal obstruction (31–40). To our knowledge, this is the first report of WE complicating a post-traumatic retroperitoneal abscess with high jejunal obstruction and pancreatitis—a unique combination of mechanical obstruction, pancreatic inflammation, and metabolic depletion. This case also demonstrates the bidirectional gut-brain axis: severe motility disorder (gastroparesis) may have been exacerbated by WE-related autonomic dysfunction, while systemic inflammation from abscess and pancreatitis likely accelerated thiamine depletion (41–43). A limitation of this case report is that standardized functional or quality-of-life scores were not used during follow-up, although the patient's clinical recovery and functional status in daily activities were clearly documented.

## Conclusion

In patients with prolonged fasting following traumatic duodenal or jejunal injury, a combined retroperitoneal and intra-abdominal abscess may cause persistent high intestinal obstruction requiring extended conservative management. Wernicke's encephalopathy should be suspected in any patient with prolonged fasting who develops unexplained neurological or autonomic symptoms, regardless of a history of alcohol use. Early brain MRI is diagnostic, and high-dose parenteral thiamine should be initiated immediately upon suspicion. Multidisciplinary collaboration among surgeons, gastroenterologists, intensivists, and neurologists is essential for managing the mechanical, inflammatory, and metabolic components of such complex cases. This case highlights that timely recognition and treatment of Wernicke's encephalopathy can lead to complete neurological recovery, even in the setting of protracted post-traumatic complications.

## Data Availability

The original contributions presented in the study are included in the article/supplementary material, further inquiries can be directed to the corresponding author.

## References

[1] Galloway PJ. Wernicke's encephalopathy and hyperemesis gravidarum. BMJ. (1992) 305:1096. doi: 10.1136/bmj.305.6861.1096-a 1467707 PMC1883622

[2] Ahmed N Vernick JJ. Pancreatic injury. South Med J. (2009) 102:1253–6. doi: 10.1097/SMJ.0b013e3181c0dfca 20016434

[3] Sharbidre KG Galgano SJ Morgan DE. Traumatic pancreatitis. Abdom Radiol. (2020) 45:1265–76. doi: 10.1007/s00261-019-02241-7 31576413

[4] Debi U Kaur R Prasad KK Sinha SK Sinha A Singh K. Pancreatic trauma: a concise review. World J Gastroenterol. (2013) 19:9003–11. doi: 10.3748/wjg.v19.i47.9003 24379625 PMC3870553

[5] Wright MJ Stanski C. Blunt pancreatic trauma: a difficult injury. South Med J. (2000) 93:383–5. doi: 10.1097/00007611-200004000-00006 10798506

[6] Onder A Kapan M Tekbas G Arikanoglu Z Aliosmanoglu I Taskesen F . Blunt trauma - related isolated pancreatic injury. Bratisl Lek Listy. (2013) 114:519–22. doi: 10.4149/BLL_2013_108 24020708

[7] Verhagen TO Aanen I van den As S de Wilde RF Allema JH Cetin F. Isolated pancreatic injury after blunt abdominal trauma: severe pathology in initially asymptomatic patient. Ned Tijdschr Geneeskd. (2024) 168:D7705. 39132882

[8] Ionescu G Szabó I Pereni O Ganea-Roth A. [Post-traumatic acute pancreatitis]. Rev Chir Oncol Radiol O R L Oftalmol Stomatol Chir. (1982) 31:417–22. 6188191

[9] Gupta V Singh Sodha V Kumar N Gupta V Pate R Chandra A. Missed pancreatic injury in patients undergoing conservative management of blunt abdominal trauma: causes, sequelae and management. Turk J Surg. (2021) 37:286–93. doi: 10.47717/turkjsurg.2021.5425 35112064 PMC8776419

[10] Altemeier WA Alexander JW. Retroperitoneal abscess. Arch Surg. (1961) 83:512–24. doi: 10.1001/archsurg.1961.01300160024004 13860719

[11] Oluwole SF Adekunle A Akintan B. Retroperitoneal abscess. J Natl Med Assoc. (1983) 75:693–700. 6887273 PMC2561484

[12] Winter BM Gajda M Grimm MO. [Diagnosis and treatment of retroperitoneal abscesses]. Urologe A. (2016) 55:741–7. doi: 10.1007/s00120-016-0118-1 27220893

[13] Li Z Tang Y Wang P Ren J. Diagnosis and Treatment of Retroperitoneal Infection. Surg Infect. (2021) 22:477–84. doi: 10.1089/sur.2020.126 33146587

[14] Altemeier WA Culbertson WR Fullen WD Shook CD. Intra-abdominal abscesses. Am J Surg. (1973) 125:70–9. doi: 10.1016/0002-9610(73)90010-X 4566907

[15] Tunuguntla A Raza R Hudgins L. Diagnostic and therapeutic difficulties in retroperitoneal abscess. South Med J. (2004) 97:1107–9. doi: 10.1097/01.SMJ.0000136264.83189.10 15586603

[16] Heye N Terstegge K Sirtl C McMonagle U Schreiber K Meyer-Gessner M. Wernicke's encephalopathy–causes to consider. Intensive Care Med. (1994) 20:282–6. doi: 10.1007/BF01708966 8046122

[17] Bolam RF. Traumatic acute pancreatitis as a cause of large bowel obstruction. Postgrad Med J. (1960) 36:53–4. doi: 10.1136/pgmj.36.411.53 13802275 PMC2481589

[18] Lee YD Lee SO Lee ST. Wernicke's encephalopathy: a complication of acute pancreatitis? Hosp Med. (2003) 64:372–3. doi: 10.12968/hosp.2003.64.6.372 12833836

[19] Arana-Guajardo AC Cámara-Lemarroy CR Rendón-Ramírez EJ Jáquez-Quintana JO Góngora-Rivera JF Galarza-Delgado DA. Wernicke encephalopathy presenting in a patient with severe acute pancreatitis. Jop. (2012) 13:104–7. 22233960

[20] Bárcena Llona J Zarranz Imirizaldu JJ. [Wernicke's encephalopathy: a complication of parenteral nutrition]. Med Clin. (1983) 81:766–8. 6418992

[21] Cardinale M Faivre A La Folie T Romand F Alla P. [Wernicke's encephalopathy following prolonged parenteral nutrition]. Presse Med. (2010) 39:1001–2. doi: 10.1016/j.lpm.2009.11.018 20430570

[22] Zeljko K Darija VB Dina LK Marko B. Wernicke's encephalopathy during parenteral nutrition in a Crohn's disease patient. Nutrition. (2011) 27:503–4. doi: 10.1016/j.nut.2010.12.005 21392706

[23] Sechi G Serra A. Wernicke's encephalopathy: new clinical settings and recent advances in diagnosis and management. Lancet Neurol. (2007) 6:442–55. doi: 10.1016/S1474-4422(07)70104-7 17434099

[24] Reuler JB Girard DE Cooney TG. Current concepts. Wernicke's encephalopathy N Engl J Med. (1985) 312:1035–9. doi: 10.1056/NEJM198504183121606 3885034

[25] Maeda H Kuriyama A Tanaka T. Prolonged hypotension associated with Wernicke's encephalopathy. Eur J Clin Nutr. (2018) 72:168–9. doi: 10.1038/ejcn.2017.141 28901332

[26] Ploner M Schnitzler A. Wernicke's encephalopathy. Lancet. (2003) 361:1000. doi: 10.1016/S0140-6736(03)12826-7 12660057

[27] Ramulu P Moghekar A Chaudhry V Zee DS Reich SG. Wernicke's encephalopathy. Neurology. (2002) 59:846. doi: 10.1212/WNL.59.6.846 12297564

[28] Watson WD Verma A Lenart MJ Quast TM Gauerke SJ McKenna GJ . in acute Wernicke's encephalopathy. Neurology. (2003) 61:527. doi: 10.1212/WNL.61.4.527 12939428

[29] Cantu-Weinstein A Branning R Alamir M Weleff J Do M Nero N . Diagnosis and treatment of Wernicke's encephalopathy: a systematic literature review. Gen Hosp Psychiatry. (2024) 87:48–59. doi: 10.1016/j.genhosppsych.2024.01.005 38306946

[30] Mayes GA. Thiamine for prevention of Wernicke's encephalopathy: a reminder. Anesth Analg. (1989) 69:407–8. doi: 10.1213/00000539-198909000-00028 2774242

[31] Makarewicz W Kaska L Kobiela J Stefaniak T Krajewski J Stankiewicz M . Wernicke's syndrome after sleeve gastrectomy. Obes Surg. (2007) 17:704–6. doi: 10.1007/s11695-007-9114-2 17658035

[32] Seehra H MacDermott N Lascelles RG Taylor TV. Wernicke's encephalopathy after vertical banded gastroplasty for morbid obesity. BMJ. (1996) 312:434. doi: 10.1136/bmj.312.7028.434 8601118 PMC2350056

[33] Truong J Shalchian S Myressiotis S Maertens de. Noordhout A, Fumal A. About a case of Wernicke's encephalopathy after sleeve gastrectomy. Eat Weight Disord. (2016) 21:721–3. doi: 10.1007/s40519-016-0318-0 27722872

[34] Sechi G. Prognosis and therapy of Wernicke's encephalopathy after obesity surgery. Am J Gastroenterol. (2008) 103:3219. doi: 10.1111/j.1572-0241.2008.02161_19.x 19086986

[35] Iannelli A Addeo P Novellas S Gugenheim J. Wernicke's encephalopathy after laparoscopic Roux-en-Y gastric bypass: a misdiagnosed complication. Obes Surg. (2010) 20:1594–6. doi: 10.1007/s11695-010-0116-0 20195787

[36] Oczkowski WJ Kertesz A. Wernicke's encephalopathy after gastroplasty for morbid obesity. Neurology. (1985) 35:99–101. doi: 10.1212/WNL.35.1.99 3966009

[37] Solá E Morillas C Garzón S Ferrer JM Martín J Hernández-Mijares A. Rapid onset of Wernicke's encephalopathy following gastric restrictive surgery. Obes Surg. (2003) 13:661–2. doi: 10.1381/096089203322190934 12935373

[38] Sánchez Rodríguez T García Marín A Camarero Mulas C Sanz Sánchez M Turégano Fuentes F. [Wernicke s encephalopathy after gastrectomy due to thiamine deficiency]. Rev Esp Enferm Dig. (2009) 101:299–300. doi: 10.4321/S1130-01082009000400014 19492910

[39] Kudru CU Nagiri SK Rao S. Wernicke's encephalopathy in a patient with gastric carcinoma: a diagnosis not to miss. BMJ Case Rep. (2014) 2014:203511. doi: 10.1136/bcr-2013-203511 24654252 PMC3962931

[40] Sinha S Kataria A Kolla BP Thusius N Loukianova LL. Wernicke encephalopathy-clinical pearls. Mayo Clin Proc. (2019) 94:1065–72. doi: 10.1016/j.mayocp.2019.02.018 31171116

[41] Belsky JB Wira CR Jacob V Sather JE Lee PJ A. review of micronutrients in sepsis: the role of thiamine, l-carnitine, vitamin C, selenium and vitamin D. Nutr Res Rev. (2018) 31:281–90. doi: 10.1017/S0954422418000124 29984680

[42] Novoa J Hardy G Manzanares W. Thiamine pharmaconutrition in sepsis: monotherapy, combined therapy, or neither? Curr Evid Safety Effic Nutr. (2023) 109:112000. doi: 10.1016/j.nut.2023.112000 36913862

[43] Kitamura K Takahashi T Tanaka H Shimotsuma M Hagiwara A Yamaguchi T . Two cases of thiamine deficiency-induced lactic acidosis during total parenteral nutrition. Tohoku J Exp Med. (1993) 171:129–33. doi: 10.1620/tjem.171.129 8128481

